# A Baseline Measurement of Quality of Life in 322 Adults With Osteogenesis Imperfecta

**DOI:** 10.1002/jbm4.10416

**Published:** 2020-11-07

**Authors:** Koert Gooijer, Arjan G J Harsevoort, Fleur S van Dijk, Hendrikje (Rik) Withaar, Guus J M Janus, Anton A M Franken

**Affiliations:** ^1^ Expert Center for adults with Osteogenesis Imperfecta Isala Hospital Zwolle The Netherlands; ^2^ Department of Medical Psychology Isala Hospital Zwolle The Netherlands; ^3^ North West Thames Regional Genetics Service Ehlers‐Danlos Syndrome National Diagnostic Service London, North West Health Care University NHS Trust Harrow UK

**Keywords:** OSTEOGENESIS IMPERFECTA, BONE DISEASE SCREENING, BONE DISEASE EPIDEMIOLOGY, PSYCHOSOCIAL ASPECTS OF OSTEOGENESIS IMPERFECTA, COLLAGEN, BONE MATRIX, AGING

## Abstract

Osteogenesis imperfecta (OI) is characterized by bone fragility and secondary features such as blue sclerae, dentinogenesis imperfecta, hearing loss, ligamentous laxity, and short stature. It was thought that health‐related quality of life (QoL) in patients with OI mainly depends on the severity of the skeletal deformities. However, it has become clear that additional factors can affect the QoL in all patients with OI. In this study, we compare dimensions of QoL in adults with OI with a control population. The SF‐36 questionnaire was distributed among 330 adult patients with different OI types. Results were compared with two control populations from the Netherlands. Age‐matched comparisons were made with one of the two control populations. The results were summarized in eight domains: general and mental health, physical and social function, bodily pain, vitality, and physical and emotional role. General health and physical function in all types of OI are low compared with controls, except patients with OI type 4 aged 55+ years. Bodily pain in patients with OI appeared significantly worse than in the control population. There was no significant difference between OI types regarding pain and vitality. Vitality was only in the OI type 1 group significantly lower compared with controls. Patients with OI type 1 had a significantly reduced mental health. Social functioning appeared most effective in type 3 around 20 years of age. QoL in adult patients with OI should be an important outcome measure in every OI clinic, but the amount of baseline data on this subject is sparse. This baseline measurement study is the largest study to date investigating QoL in adult patients with OI. The mean scores indicate that people with OI generally have a significantly lower QoL than the control population. Further qualitative evaluation of QoL and its influences is important for future management. © 2020 The Authors. *JBMR Plus* published by Wiley Periodicals LLC on behalf of American Society for Bone and Mineral Research.

## Introduction

Osteogenesis imperfecta (OI) is an inherited connective tissue disorder primarily characterized by susceptibility to fractures. The prevalence of OI has been reported to be 6 to 7 individuals per 100,000 population.^(^ ^1^ ^)^ OI is a clinically and genetic heterogeneous disorder. Clinically, OI is classified in five types (OI types 1 to 5).^(^ ^2^ ^)^ According to the clinical severity and characteristics, OI is further classified into five subtypes: nondeforming OI with blue sclerae (type 1), perinatally lethal OI (type 2), progressively deforming OI (type 3), common variable OI (type 4), and finally OI with calcification in the interosseous membranes (type 5).^(^ ^2^ ^)^ Patients can have blue sclerae, dentinogenesis imperfecta, hearing loss, joint hypermobility, and short stature as secondary features.^(^ ^3^ ^)^ Symptoms such as hearing loss, physical restrictions caused by pain, bone deformation as a result of (recurrent) fractures can increase in severity with age and can affect the health‐related quality of life (QoL) in patients with OI.

No cure for OI exists; treatment focuses on management of symptoms. Orthopedic and fracture treatment, physical therapy, special dental care, treatment for hearing loss, and medical treatment for low BMD are common therapies. However, there has been less attention paid to the psychosocial impact of living with OI in adults.

Today, it is commonly recognized that measuring the QoL in people with OI can provide new information to improve treatment and subsequently the QoL of patients. Here, we report on the QoL of 322 patients with a diagnosis of OI type 1, 3, and 4 in the Netherlands compared with the general Dutch population. We suspected that the QoL in patients with OI would be decreased compared with controls. To measure the QoL in a patient cohort with OI, we decided to use the validated self‐reported health assessment tool, the SF‐36 questionnaire,^(^ ^5^, ^6^ ^)^ which is frequently used in international studies. The SF‐36 measures QoL across eight different subscales. We compared the SF‐36 subscales against the different OI‐type groups and with the QoL data of two Dutch control groups, including different age categories.

## Patients and Methods

### Study design and population

A cross‐sectional cohort study was undertaken in the National Expert Center for Adults with Osteogenesis Imperfecta, Isala Hospital, Zwolle, the Netherlands. In this center, patients with a clinical and usually confirmed molecular diagnosis of OI are assessed by the multidisciplinary OI team. The SF‐36 questionnaire^(^ ^4^ ^)^was provided during the first appointment. All new adult patients who attended the center from December 2007 until November 2018 were selected. Exclusion criteria were age <18 years and unavailability to fill in the questionnaire. Informed consent was obtained from each participant, and the Medical Ethics Committee of the Isala Hospital, Zwolle, the Netherlands, approved the study protocol and provided a non‐WMO (Medical Research Involving Human Subjects Act) waiver.

### Evaluation of quality of life in patients with OI

QoL was assessed using the validated self‐reported health assessment tool, the SF‐36 questionnaire,^5^, ^6^ ^)^ which is composed of 36 questions in eight different domains that examine aspects of physical and mental health in a 4‐week timeframe. The SF‐36 questionnaire is used in multiple countries to measure QoL in patients; it has been extensively tested for reliability and validity.^(^ ^6^, ^7^, ^8^, ^9^, ^10^ ^)^The four main physical domains are physical function, role limitations caused by physical health problems, bodily pain, and general health perceptions. The four main mental domains are vitality, social function, role limitations based on emotional problems, and general mental health. Each domain score is linearly converted to a 0 to 100 scale. A higher score is correlated with better mental and physical health. The physical and mental domains can be summarized in two broad scores: the physical component summary and the mental component summary. These summary scores reflect self‐assessed physical and mental activity.

All patients with different types of OI were divided in age categories to compare QoL in patients with OI.

### Control groups

The control values are based on two different studies. The first control was the result of a municipal screening that was carried out in 1992 by the University of Groningen, the Netherlands. It concerned a group of 1063 adults, randomly selected from the civil register of Township Emmen. The data of this control group were available according to different age ranges.^(^ ^11^ ^)^ For the general comparison, a national randomly selected control group without age range (*n* = 1742) was used. Data from these individuals were generated from a study conducting a nationwide, population‐based health status survey for the purpose of generating normative data for a study of patients with congenital heart defects.^(^ ^5^ ^)^

**Fig 1:**
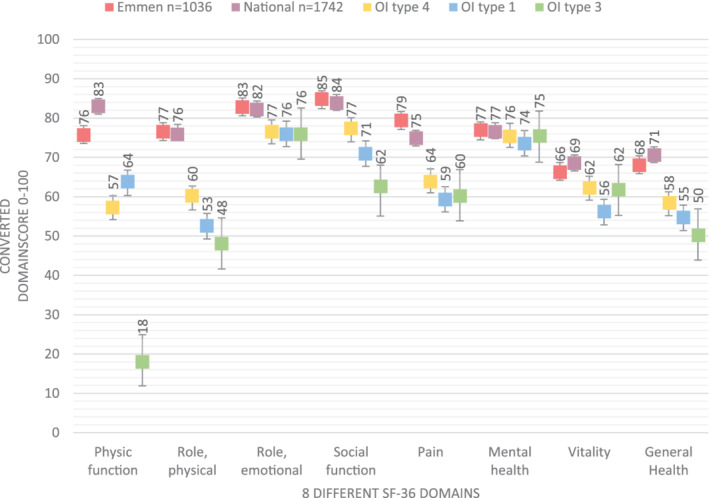
Visualization of the eight different SF‐36 questionnaire domains, divided per osteogenesis imperfecta type and control group.

The SF‐36 questionnaire results of both control groups are presented in Fig. 1.

### Data and statistical analysis

In Table 1, the data of both control groups^(^ ^5^, ^11^ ^)^ have been combined and compared with the recruited patients with OI. Only the first control group^(^ ^11^ ^)^ was used for the data presented in the Supplementary Appendix. For each age category, a comparison was made with the age‐matched control patients to test if the null‐hypothesis (no differences between OI and controls) could be rejected. Then, the OI types were reciprocally compared. To calculate a Δ score, the score of the youngest patient group was subtracted from the eldest patient group. As the oldest OI type 3 group consisted only of three individuals, the Δ score was not calculated. Given that the questionnaire score cannot be reliably estimated for participants with extreme scores, floor and ceiling effects were examined (Table 1).

**Table 1:** SF‐36 Questionnaire Scores for Patients With Osteogenesis Imperfecta According to OI Types and for the General Population

Physical domains	Osteogenesis imperfecta (OI) type								
	OI (all types) (*n* = 322)	*P* Value	Type 1 (*n* = 220)	*P* Value	Type 3 (*n* = 40)	*P* Value	Type 4 (*n* = 62)	*P* Value	General population (*n* = 1063 + 1742)
PF	57 ± 31.3	.000	63,6 ± 28,6	.000	**18.4 ± 21.8**	.000	57.2 ± 28.4	.000	79.4 ± 22.8
RP	50 (0; 100)	.000	**50 (0; 100)**	.000	**50 (0; 100)**	.000	**75 (0; 100)**	.012	76.5 ± 36.3
BP	60.4 ± 27.2	.000	59.4 ± 27.8	.000	60.4 ± 25.5	.000	**64 ± 26**	.000	77.14 ± 24.4
GH	54.8 ± 20.7	.000	54,6 ± 20,4	.000	50.4 ± 23.9	.000	58,2 ± 19,3	.000	69.42 ± 21.14
PCSS	39.6 ± 11.6	.000	40.6 ± 11.8	.000	31.8 ± 9.2	.000	41 ± 10,4	.000	50 ± 10
Mental domains									
Vitality	58 ± 20.6	.000	56.1 ± 20.5	.000	61.7 ± 20.6	.081	62.2 ± 20.4	.043	67.53 ± 19.9
SF	75 (50; 100)	.000	75 (50; 100)	.000	**62.5 (37.5; 87.5)**	.000	**75 (62.5; 100)**	.066	84 ± 22.3
RE	100 (66; 100)	.018	**100 (66; 100)**	.064	**100 (66; 100)**	.438	**100 (66; 100)**	.210	82.57 ± 33.3
MH	74.2 ± 18	.012	73.6 ± 18	.009	75.3 ± 18.9	.618	75.6 ± 17.7	.600	76.8 ± 17.56
MCSS	49.7 ± 11.2	.615	48.7 ± 10.9	.087	53.2 ± 11.2	.078	51 ± 11.6	.492	50 ± 10

Data shown as mean ± SD or median (interquartile range) as appropriate. *p* Values are osteogenesis imperfecta vs general population (summary independent *t* test or one‐sample Wilcoxon signed‐rank test). Values indicating floor (≥15% with a score of 0) and ceiling (≥15% with a score of 100) effects are in bold face.

BP = Bodily pain; GH = general health; MCSS = mental component summary score; MH = mental health; PCSS = physical component summary score; PF = physical functioning; RE = role functioning emotional; RP = role functioning physical; SF = social functioning.

Variables were tested for normal distribution with the Kolmogorov–Smirnov test, Shapiro–Wilk test, and q‐q plots. Means and SDs were given for normally distributed continuous variables. Non‐normally distributed continuous variables were presented as median, interquartile range (IQR). Differences in means comparing patients with OI with the controls were in normally distributed data tested using the summary independent sample *t* tests and in not normally distributed data tested with the one‐sample Wilcoxon signed‐rank test. Comparisons between OI types of different ages were done using ANOVA in normally distributed data, and with independent‐samples Kruskal‐Wallis tests with Dunn's comparison for post hoc testing in not normally distributed data. A two‐sided *p* value of 0.05 was considered significant. Significance values for comparison between OI types have been adjusted by the Bonferroni correction for multiple testing. Significance values for comparing patients with OI with controls are presented with three decimals for adequate interpretation. Analyses were performed using SPSS 25 (SPSS, Inc., Chicago, IL, USA) for Windows.

We did not assess separately modifiers of QoL such as fracture history, scoliosis, and pulmonary function.

## Results

### Clinical characteristics

A total of 372 patients with OI were identified for participation in the current study. Fifty patients were excluded as their SF‐36 questionnaires were unavailable. Therefore, 322 patients were available for analysis.

A total of 190 (59%) of the 322 patients with OI in our cohort were women; 132 (41%) were men. The mean and median age of participants with OI at the first visit were, respectively, 38 and 35.5 years (interquartile range [IQR] 27 years). Skewness and kurtosis were, respectively, 0.343 and −1.119, confirming a normal distribution with a small overrepresentation of the middle group.^(^ ^12^ ^)^ There were 220 (66.7%) subjects who had a diagnosis of OI type 1, 40 (12.1%) were diagnosed with OI type 3, and 61 patients (18.5%) had OI type 4.

### Scores of all patients with OI across eight different SF‐36 subscales

Figure 1 shows the results of the eight different SF‐36 subscales of the three OI types in comparison with two control groups in the Netherlands.^(^ ^5^, ^11^ ^)^

Individuals with OI type 1, 3 and 4 had a significantly lower mean physical function score compared with the control groups (Table 1).^(^ ^5^, ^11^ ^)^ A significant difference between patients with OI and controls applied to all the subscales except for vitality in OI type 3, role limitations caused by emotional problems in OI types 3 and 4, and mental health in OI types 3 and 4 (Table 1).

### Comparison of SF‐36 subscale scores in different age categories

A complete overview of the results is available in the Supplementary Table S2. The comparisons have been made with the first control group^(^ ^11^ ^)^ because in this control group participants were divided in age categories, suitable for making age‐matched comparisons.

#### Physical functioning

Physical function in the overall OI cohort was significantly lower compared with controls^(^ ^5^, ^11^ ^)^ in all different age categories, except for patients with OI type 4 and aged >55 years (Table 1 and Supplementary Table S2).^(^ ^11^ ^)^ Individuals with OI type 3 had the lowest score on physical function, (Table 1), whereas individuals with OI type 1 in the age group 18 to 24 years had the highest score on physical function. The physical function of OI type 3 was significantly lower than OI type 1 and 4 in all age categories except when compared with OI type 4 in the age group 35 to 54 years. (Supplementary Table S2). The physical function of individuals with OI types 1 and 4 in the different age categories were not significantly different from each other, except in the age category 18 to 24 years (Supplementary Table S2).

In the control group,^(^ ^11^ ^)^ physical function declined during at least 30 years with 27.9 points (Δ; see Patients and Method section). The OI type 1 group followed that trend (Δ −21.72, *p* < 0,05), whereas patients with OI type 4 showed a climbing trend (Δ +18.7, *p* = 0.106), implying that several individuals >55 years with OI type 4 experienced a higher physical function than patients with OI type 4 aged 18 to 24 years (Supplementary Table S1).

#### Role limitations caused by physical health problems

The role limitations caused by physical health problems in individuals with OI was significantly lower compared with controls(^(^ ^5^, ^11^ ^)^ Table 1). For patients with OI type 3, this was not significant for all age categories from 25 to 55+ years. For patients with OI type 4, this was not significant above the age of 35 years. Between OI types, there was a less‐significant difference at different ages (Supplementary Table S2). Patients with OI type 4 aged 35 to 54 years (Supplementary Table S1) had the lowest score on role limitations caused by physical health problems. OI type 3 patients had the lowest score on role limitations caused by physical health problems, but the score increases until their mid‐50s when the difference between OI type 3 and the controls^(^ ^11^ ^)^ was not significant anymore (Supplementary Table S2).

The control group^(^ ^11^ ^)^ trend over the years was decreasing slowly (Δ −15.4; Table 1). The OI type 1 group had a comparable trend (Δ –11.3), whereas patients with OI type 4 had an increasing score over the years (OI type 4: Δ +7.5). None of the OI trend values were significant.

#### Bodily pain

All patients with OI experienced significantly more pain than the control group (Table 1),^(^ ^5^, ^11^ ^)^ also within different age categories (Supplementary Table S2). On a scale from 1 to 100 points, patients with OI scored an average of 17 points lower than the control group. There was no significant difference in pain between OI types in different age categories (Supplementary Table S2). The pain in patients with OI type 1 and type 4 was higher in the oldest patient group compared with the youngest patient group (OI type 1: Δ −12.9, *p* = 0.101; OI type 4: Δ −7.3, *p* = 0.440). This was comparable to the Δ of the control group^(^ ^11^ ^)^ (Δ −13.1). The lowest score on bodily pain was reported in patients with OI type 4 between the ages of 25 and 34 years (Supplementary Table S1).

#### General health perceptions

Across all the age categories, except for people with OI type 4 > 55 years, patients with OI had a significant lower general health than their controls^(^ ^11^ ^)^ (Supplementary Table S2). There was no significant difference in health perceptions between OI types in all age categories. Health perception of patients with OI decreased less from the youngest to the eldest age group compared with healthy people^(^ ^11^ ^)^ (Δ mean OI: –5.22, Δ mean controls: −15.88). This resulted in a statistically nonsignificant difference of health perception between patients with OI and controls in the age category of 55+ years.

#### Vitality

Only patients with OI type 1 showed a significantly lower vitality compared with the control group^(^ ^5^, ^11^ ^)^ (Table 1), except when >55 years^(^ ^11^ ^)^ (Supplementary Table S2). Patients with OI types 3 and 4 did not have significantly reduced vitality compared with controls,^(^ ^11^ ^)^ except for OI type 3 in the age category of 35 to 54 years. There was no significant difference in vitality between the OI types in different age categories.

Vitality in the control group^(^ ^11^ ^)^ slightly decreased from the youngest to the eldest age group (Δ −4.5). This was similar in the patients with OI type 1 (Δ −0.1) and OI type 4 (Δ −8.83; Supplementary Table S1).

#### Social functioning

Social function in patients with OI with types 1, 3, and 4 was significantly lower compared with the control group^(^ ^5^, ^11^ ^)^ (Table 1). However, when analyzed per age category these results were in some instances not significant^(^ ^11^ ^)^ (Supplementary Table S2). There was one statistically significant difference in social functioning between OI types in different age categories. In age category 18 to 25 years; people with OI type 3 scored significantly lower than people with OI type 1 and OI type 4.

#### Role limitations caused by personal or emotional problems

Role limitations caused by personal of emotional problems of patients with OI in general were not statistically different from the control population.^(^ ^5^, ^11^ ^)^ Regarding the different age categories, there was a statistically significant difference between patients with OI type 4 and controls in the age category 18 to 24 years.^(^ ^11^ ^)^

There was no significant difference regarding role limitations between the OI types in all age categories. Individuals with OI type 4 scored lowest regarding role limitations in the age category 25 to 34 years (Supplementary Table S1). Patients with OI type 4, aged 19 to 24 years, scored highest, even significantly higher than the control group.^(^ ^11^ ^)^

#### Mental health

We observed—only in patients with OI type 1—a very small, but significantly reduced mental health compared with controls^(^ ^5^, ^11^ ^)^ (Table 1). When analyzing specific age categories in patients with OI type 1, patients with OI aged 35 to 54 years had a significant reduced mental health (Supplementary Tables S1 and S2). There was no significant difference between the OI types. The oldest and the youngest groups had similar outcomes.

## Discussion

Most studies on QoL in OI have focused on children; hence, studies reporting on QoL of adult patients with OI are sparse. We used the SF‐36 questionnaire to measure QoL in 322 adults with OI. The objective was to describe and compare the QoL in adults with a clinical diagnosis of OI types 1, 3, and 4 in different age categories with controls. The control group consisted of 2834 healthy Dutch adults reported in two studies,^(^ ^5^, ^11^ ^)^ with one group divided into age categories (*n* = 1063).^(^ ^11^ ^)^

A recent online survey of 300 self‐reported patients with OI, consisting of 198 adults, investigated QoL using nine patient‐reported outcomes measurement information system (PROMIS) computer adaptive testing (CAT) instruments.^(^ ^13^ ^)^ QoL has also been investigated in adults with OI using the SF‐36 questionnaire. In these studies, the number of adult participants ranged from 15 to 85,^(^ ^14^, ^15^, ^16^, ^17^, ^18^ ^)^ which makes the current study the largest study to date investigating QoL in adults with OI.

Our adult OI cohort reported significantly decreased psychosocial and physical QoL across multiple domains and age groups, compared with the control group(s). We identified multiple significant differences between adults with OI and the controls.

The results of physical function per OI type and age category reflect what we see in our outpatient clinics. Physical function in the overall cohort is significantly lower compared with controls, and patients with OI type 3 have the lowest physical function. Patients with OI type 1 aged 18 to 24 years have the highest physical function. This may be because it is OI type 1, which is characterized by the absence of bone deformation, and sometimes it can be mild and difficult to diagnose in the absence of a family history. Additionally, in adulthood, the fracture rate is known to decrease significantly in contrast to the childhood fracture rate. Only in patients with OI type 1 did we observe significantly reduced mental health compared with controls, probably because of the greater sample size. Mental health in the overall OI cohort compared with the control groups was significantly lower and in line with observations by Hald and colleagues^(^ ^18^ ^)^ and Widmann and colleagues,^(^ ^15^ ^)^ where the mental domains were less affected than the physical domains in people with OI. Supplementary Table S3 provides a detailed comparison with only the study by Hald and colleagues because of their larger number of participants (*n* = 85) and data availability.

The relative sparing of psychosocial dimensions of QoL in patients with OI was also observed in patients with Marfan syndrome^(^ ^19^ ^)^and patients living with congenital heart disease,^(^ ^20^ ^)^ as well as patients with OI.^(^ ^18^ ^)^ Perhaps the adults with OI have developed coping skills during their childhood that allow for normal psychosocial functioning despite their physical limitations.

The difference between physical severity measured by physical function and subjective severity perception measured by general health perception illustrates that patients may perceive the disorder differently from health care professionals. This is important for health care providers to acknowledge when discussing patient reported symptoms in clinical practice. Patient reported QoL should be incorporated into clinical practice to ensure the patient's perspective is included in clinical decision‐making. The mean pain in patients with OI is significantly increased compared with the control group, but between the OI types there are no significant differences. The presence of pain would imply a more‐severe disease, but there is no evident association between pain and OI type in our cohort. This is comparable with observations of other studies,^(^ ^15^, ^21^ ^)^ and has also been observed in review studies for pain in children with OI.^(^ ^22^ ^)^

Vitality in patients with OI is only slightly lower than the control group. Some studies reported diminished vitality and social functioning abilities^(^ ^23^ ^)^ with reduced mental health and emotional functioning compared with the adult control group.^(^ ^17^ ^)^ In our study, only for patients with OI type 1 is vitality significantly lower than in the controls. This is important to know when seeing patients with OI with complaints about reduced vitality: Other possible causes should be excluded first, and reduced vitality should not immediately be assumed to be a feature of patients with OI.

In our cohort, there is no significant difference in social functioning between patients with OI type 1 and type 4 after the age of 25 years. There seems to be reduced social functioning in patients with OI type 3 under the age of 25 years. This improves around 25 years of age. A possible explanation could be a transitional phase where patients are becoming independent, must handle problems themselves, and acquire better social functioning skills. The large role of caretakers in daily care, frequent health care appointments, and the effort required to stay safe^(^ ^24^, ^25^, ^26^, ^27^, ^28^, ^29^ ^)^ are increasingly transitioned to the adults with OI giving them more control. Also, a decline in fracture rate in adults with OI compared with children with OI can play a role.

### Influencing the quality of life

This study provides a baseline measurement of QoL in adults with OI. It is no surprise that the overall QoL in patients with OI is significantly lower at all age ranges and in all OI types compared with the control group. However, this baseline measurement is important because it signals which components are most affected in which health domain in which OI type at what age. It does not provide an answer for the question regarding factors that influence the different health domains of QoL, which is why specifically designed questionnaires focused on determining factors of QoL in adults with OI are essential to improving QoL and are currently being developed.

Identifying specific outcomes that are associated with improved or decreased QoL in OI is important to guide timing and nature of interventions and to design research aimed at optimizing well‐being of adults with OI.^(^ ^21^, ^30^ ^)^ For example, Dahan‐Oliel and colleagues^(^ ^21^ ^)^ performed a systemic review of previously mentioned studies^(^ ^14^, ^15^, ^16^, ^17^ ^)^ and concluded that for both children and adults with OI pain, scoliosis, activity limitations, and participation restrictions caused by decreased limited function are associated with lower levels of physical QoL and need to be addressed to promote QoL.

When interventions are planned, a follow‐up measurement of QoL can indicate the effect of these interventions on the different health domains and as such, the impact of these interventions can be measured.

In our adult OI service we have tried to identify factors that might positively influence the QoL in people with OI. For this purpose, a value‐based health care program has been developed to identify factors that we can influence in our service and that are measurable by QoL questionnaires. These aspects would be consistently monitored through the years. A very important influence on the development of a value‐based health care program is the input of the OI group regarding what they consider important for their QoL.

### Limitations and future plan

This study reports on baseline measurements of the QoL in 322 adult patients with OI measured by the SF‐36 questionnaire. The SF‐36 is a generalized, QoL questionnaire that is not specific for people with OI. This makes the data susceptible to temporary biases such as a recent fracture. However, the SF‐36 is well‐validated and widely used; therefore, it is a valid tool to evaluate QoL for patients with OI. As mentioned earlier, the development of OI‐specific questionnaires is important and in progress; the results of this study can serve as basis for their development.

We compared our patient data against the data of reference populations collected more than two decades ago. Nonetheless, the reference populations were unique and representative of the Dutch population. The SF‐36 is sensitive to fluctuations in health,^(^ ^4^ ^)^ which makes it suitable to measure QoL over a longer period or before and after a procedure. As such, we will aim to present a longitudinal overview of QoL in patients with OI through measurements of QoL and its influences at different time points.

## Conclusion

Our study described baseline QoL measurements in the largest group of adults with different types of OI to date (*n* = 322) and compared outcomes with (age‐matched) control groups. The mean scores indicated that people with OI generally had a significantly lower QoL than the control population, and the scores per domain gave insight into which domains at what age in which OI type were more severely affected. This is important information for aging patients with OI and their health care professionals. Longitudinal QoL measurement and further qualitative evaluation of QoL and its influences are important for future management and improvement of QoL in people with OI.

## Disclosure

The authors declare that they have no conflicts of interests.

## AUTHOR CONTRIBUTIONS

**K Gooijer:** Conceptualization; data curation; formal analysis; investigation; methodology; visualization; writing‐original draft. **Arjan Harsevoort:** Conceptualization; data curation; methodology; resources; writing‐review and editing. **Fleur Dijk:** Conceptualization; methodology; writing‐review and editing. **Rik Withaar:** Methodology; validation; writing‐review and editing. **Guus Janus:** Conceptualization; project administration; writing‐review and editing. **Anton Franken:** Conceptualization; project administration; supervision; writing‐review and editing.

## Availability of Data and Material

The data sets used and analyzed during the current study are available from the corresponding author on reasonable request.

## Supporting information


**Appendix S1.** Supporting Information.

**Supplementary Table S3**. Comparison of Current study with the study of Hald et al. 2017 per type of OI
