# Supplementary material for: A Baseline Measurement of Quality of Life in 322 Adults With Osteogenesis Imperfecta
Source: JBMR Plus. 2020 Nov 7;4(12):e10416. doi: 10.1002/jbm4.10416 (PMC7745883; doi:10.1002/jbm4.10416)
Supplement: Supplementary file 2 — Supplementary Table S3. Comparison of Current study with the study of Hald et al. 2017 per type of OI [file JBM4-4-e10416-s002.docx]

Table 3 Appendix. Comparison of Current study with the study of Hald et al. 2017 per type of OI

|  | Current study | Hald et al. 2017 | P | Current study | Hald et al. 2017 | P | Current study | Hald et al. 2017 | P |
| --- | --- | --- | --- | --- | --- | --- | --- | --- | --- |
|  | OI type 1 (n=220) | OI type 1 (n=58) |  | OI type 3 (n=40) | OI type 3 (n=11) |  | OI type 4 (n=62) | OI type 4 (n=15) |  |
|  | Mean±SD | Mean±SD |  | Mean±SD | Mean±SD |  | Mean±SD | Mean±SD |  |
| Physical Function | 63,6±28,6 | 72.8±26.4 | 0.023 | 18,4±21,8 | 20.9±21.5 | 0.737 | 57,2±28,4 | 64.7±30.4 | 0.368 |
| Role function Physical | 52,5±43,1 | n/a | n/a | 48,1±45,1 | n/a | n/a | 59,7±42,8 | n/a | n/a |
| Bodily Pain | 59,4±27,8 | 59.3±28.4 | 0.981 | 60,4±25,5 | 60.6±28.6 | 0.991 | 64±26 | 64±27.2 | 1.000 |
| General Health | 54,6±20,4 | 60.4±22.5 | 0.061 | 50,4±23,9 | 63.1±24.3 | 0.126 | 58,2±19,3 | 55.5±24.8 | 0.648 |
| Physical Component Summary Score | 40,6±11,8 | 40.5±11.5 | 0.954 | 31,8±9,2 | 30.5±9.4 | 0.681 | 41±10,4 | 39.2±11.5 | 0.557 |
| Vitality | 56,1±20,5 | 59.3±24.9 | 0.370 | 61,7±20,6 | 63.2±22.6 | 0.835 | 62,2±20,4 | 55.3±27.4 | 0.277 |
| Social Function | 70±27,8 | n/a | n/a | 61,6±29,6 | n/a | n/a | 77±25 | n/a | n/a |
| Role function Emotional | 75±39,1 | n/a | n/a | 76,1±35 | n/a | n/a | 76,5±17,7 | n/a | n/a |
| Mental Health | 73,6±18 | 79.9±15.9 | 0.016 | 75,3±18,9 | 82.2±10.3 | 0.120 | 75,6±17,7 | 78.9±21.5 | 0.537 |
| Mental Component Summary Score | 48,7±10,9 | 52.7±10.3 | 0.013 | 53,2±11,2 | 60.6±8.7 | 0.048 | 51±11,6 | 53±12.5 | 0.557 |
